# Non-immune functions of B7-H3: bridging tumor cells and the tumor vasculature

**DOI:** 10.3389/fonc.2024.1408051

**Published:** 2024-06-17

**Authors:** Shuo Wu, Chenxi Hu, Kaiyuan Hui, Xiaodong Jiang

**Affiliations:** ^1^ Department of Oncology, Lianyungang Clinical College of Nanjing Medical University, Lianyungang, China; ^2^ Department of Oncology, The Affiliated Lianyungang Hospital of Xuzhou Medical University, Lianyungang, China

**Keywords:** B7-H3, non-immune functions, tumor cells, tumor vasculature, cancer therapy

## Abstract

B7-H3 (CD276), an immune checkpoint molecule, is overexpressed in various types of cancer and their tumor vasculature, demonstrating significant associations with adverse clinical outcomes. In addition to its well-known immune functions, B7-H3 exhibits dual co-stimulatory/co-inhibitory roles in normal physiology and the tumor microenvironment. The non-immune functions of B7-H3 in tumor cells and the tumor vasculature, including promoting tumor cell anti-apoptosis, proliferation, invasion, migration, drug resistance, radioresistance, as well as affecting cellular metabolism and angiogenesis, have increasingly gained attention from researchers. Particularly, the co-expression of B7-H3 in both tumor cells and tumor endothelial cells highlights the higher potential and clinical utility of therapeutic strategies targeting B7-H3. This review aims to summarize the recent advances in understanding the non-immune functions of B7-H3 in tumors and provide insights into therapeutic approaches targeting B7-H3, focusing on its co-expression in tumor cells and endothelial cells. The aim is to establish a theoretical foundation and practical reference for the development and optimization of B7-H3-targeted therapies.

## 1 Introduction

CD276, also known as B7 homolog 3 (B7-H3), is a type I transmembrane protein consisting of 316 amino acids. It is encoded by the gene located on chromosome 9 in mice and the human 15q24 region. The primary structure of B7-H3 includes an extracellular Ig-like domain, a transmembrane region, and a short cytoplasmic tail (1). Based on the number of extracellular Ig-like domains, membrane-bound B7-H3 can be classified into two isoforms: 2IgB7-H3 and 4IgB7-H3. The former contains one IgV (variable) and one IgC (constant) domain, while the latter possesses tandem IgV and IgC domains due to exon duplication (2).

B7-H3 is a cell surface tumor endothelial marker with up to 30% amino acid homology to other members of the B7 family. It is also expressed in tumor-associated endothelial cells and is often associated with advanced tumor staging (3). B7-H3 has been shown to differentiate pathological and physiological angiogenesis in both mice and humans (4). The high expression of B7-H3 protein is observed in various human cancers such as lung, breast, colon, endometrial, renal, and ovarian cancer tumor vasculature, while it is not expressed in normal ovarian vasculature (5).

**Figure 1:**
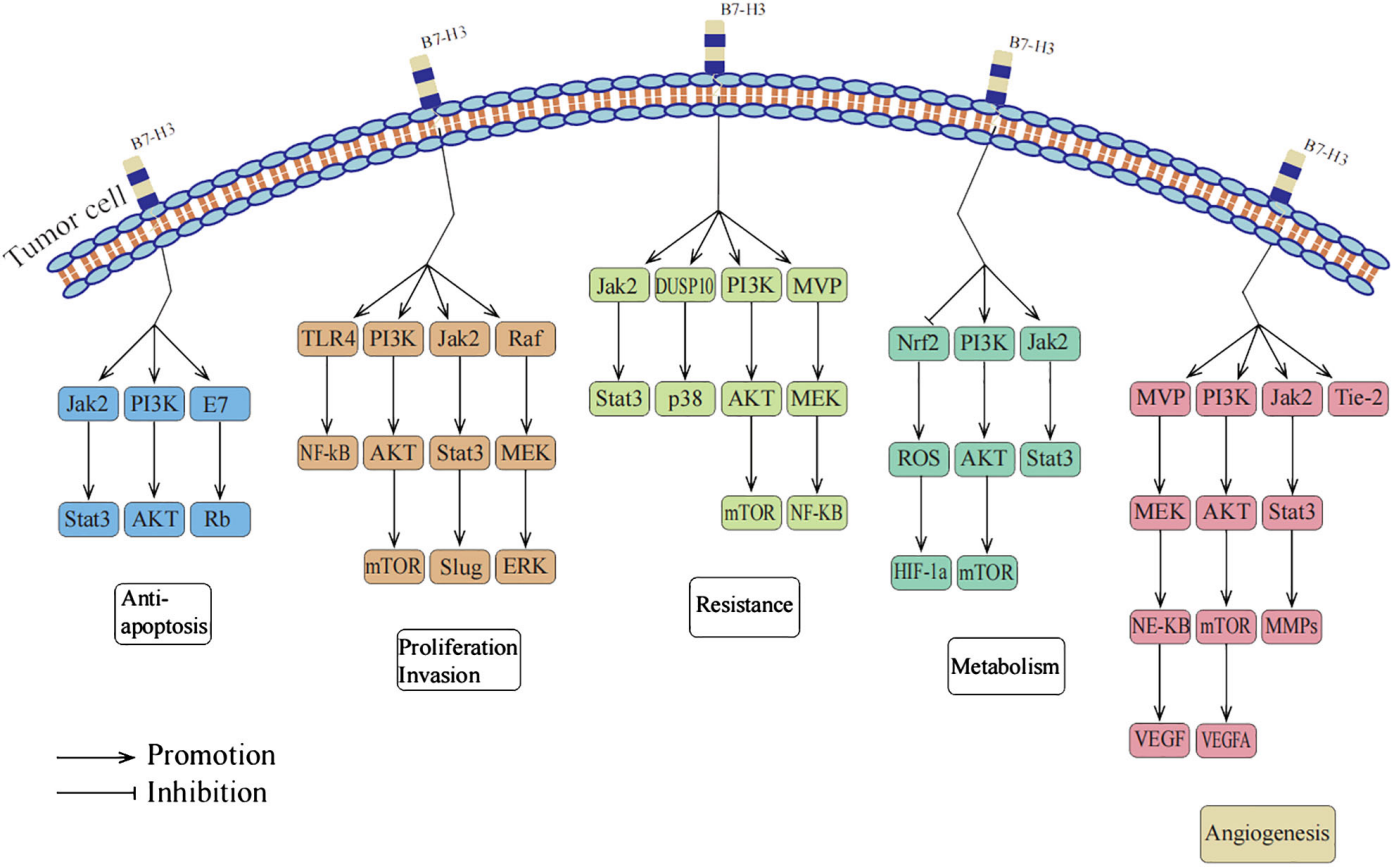
B7-H3 promotes tumor progression through non-immune functions.

Although initially characterized as a T-cell co-stimulatory protein, current research describes B7-H3 as a T-cell inhibitory molecule that promotes tumor invasion and proliferation. It mediates immune escape primarily by inhibiting T-cell infiltration and promoting CD8^+^ T-cell exhaustion, suggesting that B7-H3 may serve as an important immune target in cancer therapy (6). However, B7-H3 also functions as an independent protein outside of the immune system, participating in non-immune responses in tumors. It plays a crucial role in tumor cell anti-apoptosis, proliferation, invasion, migration, drug resistance, radioresistance, metabolism, and angiogenesis (7). The exact mechanisms through which B7-H3 promotes tumor development independently of the immune system are not fully understood. Nevertheless, extensive research suggests that B7-H3 may exert its effects upstream of signaling pathways, indicating that targeting these pathways could provide new approaches for cancer treatment (3). The non-immune functions of B7H3 promoting tumor progression are summarized in **Figure 1**.

## 2 B7-H3 in the non-immune functions of tumors

### 2.1 Anti-apoptosis

Apoptosis is a genetically regulated, orderly process of cell death primarily responsible for eliminating aged or abnormal cells. Resistance to apoptosis has been widely recognized as a crucial hallmark of malignant tumors (8). B7-H3 promotes tumor development by suppressing apoptosis in tumor cells (9). In a multi-omic analysis and single-cell sequencing study, Zhou et al. found a significant increase in B7-H3 levels during the cellular senescence process in tumor cells, suggesting a critical role of B7-H3 in preventing tumor cell senescence (10). On the other hand, the role of B7-H3 in tumor growth has also garnered attention. Early studies on breast cancer cells demonstrated that silencing B7-H3 expression in the MCF-7 cell line led to an upregulation of vascular endothelial growth factor (VEGF) at both mRNA and protein levels, indicating that B7-H3 may inhibit tumor growth by suppressing VEGF expression (11). However, subsequent research revealed that the expression of B7-H3 actually facilitates tumor growth in other breast cancer cell lines, suggesting a complex and potentially contradictory role of B7-H3 in the pathogenesis of breast cancer (12). These findings indicate that B7-H3 may play a multifaceted role in the development of tumors, with specific effects likely dependent on the tumor type, underlying mechanisms, and biological context. Nonetheless, the majority of current research supports the view that B7-H3 promotes tumor growth through its anti-apoptotic effects. The summary of signaling pathways mediating the anti-apoptotic effects of B7-H3 is presented in **Table 1**.

**Table 1:** Signaling Pathways Mediating the Diverse Functions of B7-H3.

Function	Cancer Type	Mechanism	References
Anti-apoptosis	Colorectal Cancer	Upregulates the Jak2-Stat3 signaling pathway to enhance the expression of downstream anti-apoptotic proteins Bcl-2 and Bcl-xl, thereby strengthening tumor cell resistance against apoptosis	(13)
	Colorectal Cancer	Suppresses cellular senescence in colorectal cancer cells through the AKT/TM4SF1/SIRT1 pathway	(14)
	Gastric Cancer	Inhibits tumor cell apoptosis by regulating the PI3K/AKT signaling pathway mediated by fibronectin	(8)
	Cervical Intraepithelial Neoplasia and Cervical Cancer	Induces tumor cell apoptosis by modulating the E7/Rb pathway, affecting cell cycle and apoptosis	(15)
Proliferation, Invasion, Migration	Breast Cancer	Regulates MEK to induce tumor stem cell proliferation	(16)
	Head and Neck Squamous Cell Carcinoma	Activates AP-1 to promote cancer stem cell invasion and metastasis	(17)
	Ovarian Cancer	Affects tumor cell invasion, migration, and proliferation through the Jak2/Stat3 pathway	(18)
	Gastric Cancer	Silencing B7-H3 downregulates CXCR4 related to metastasis and inhibits phosphorylation of AKT, ERK, and Jak2/Stat3, suppressing tumor cell migration and invasion	(19)
	Glioblastoma	Enhances tumor cell proliferation and invasion through the activation of Jak2/Stat3/Slug signaling pathway. Induces EMT process by downregulating E-cadherin and upregulating MMP-2/9 expression, leading to increased invasion of glioblastoma cells	(20)
	Pancreatic Ductal Adenocarcinoma	BRD4/B7-H3/TLR4 axis promotes tumor cell proliferation, invasion, and metastasis	(21)
	Liver Cancer	Targets epithelial-mesenchymal transition (EMT) through the Jak2/Stat3/Slug signaling pathway while promoting the expression of MMP-2 and MMP-9 to enhance tumor metastasis and invasion	(22)
	Non-Small Cell Lung Cancer	Upregulates SIRT1 expression through the PI3K/AKT pathway, activating EMT and inducing migration	(23)
	Melanoma	Knockdown of B7-H3 reduces MMP-2 mRNA levels and proMMP-2 protein expression, as well as decreases phosphorylation of Stat3 and secretion of IL-8, leading to decreased metastatic capability	(24)
	Breast Cancer	Accelerates lung metastasis by activating the Raf/MEK/ERK signaling pathway	(25)
	Clear Cell Renal Cell Carcinoma	Promotes tumor cell invasion and metastasis in a fibronectin-dependent manner, accompanied by upregulation of phosphorylated proteins in the PI3K/AKT and p38/ERK MAPK signaling pathways	(26)
Drug Resistance	Breast Cancer	Partially induces paclitaxel resistance by interfering with the Jak2/Stat3 pathway	(27)
	Pancreatic Cancer	Induces gemcitabine resistance through downregulation of survivin expression	(28)
	Melanoma	Mediates dacarbazine and cisplatin resistance via DUSP10-mediated p38 MAPK inactivation	(29)
	Colorectal Cancer	Upregulates expression of X-ray repair cross-complementing protein 1 (XRCC1) through the PI3K-AKT pathway, promoting oxaliplatin resistance	(30)
	Colorectal Cancer	Reduces G2/M phase arrest in a CDC25A-dependent manner, enhancing resistance to oxaliplatin or 5-fluorouracil	(31)
	Ovarian Cancer	Induces activation of the PI3K/AKT signaling pathway, leading to treatment resistance	(32)
Metabolism	Esophageal Squamous Cell Carcinoma	Induces phosphorylation of PKM2 through the Stat3 signaling pathway, promoting glucose metabolism	(33)
	Breast Cancer	Regulates glucose metabolism by inhibiting Nrf2 and its target genes, increasing protein levels of HIF1α, LDHA, and PDK1	(34)
	Colorectal Cancer	Regulates glucose metabolism by inhibiting Nrf2 and its target genes, increasing protein levels of HIF1α, LDHA, and PDK1	(34)
	Oral Squamous Cell Carcinoma	Enhances glycolysis by upregulating Glut1 and PFKFB3 through the PI3K/AKT/mTOR/HIF-1α pathway	(35)
	Colorectal Cancer	Regulates glucose metabolism by controlling the expression of HK2	(36)
	Neuroblastoma	Increases glucose uptake and lactate production by regulating the Stat3/c-Met pathway and increasing PFKFB3 expression	(37)
Angiogenesis	Neuroblastoma	Induces angiogenesis through MMP-2 and MMP-9	(38)
	Colorectal Cancer	Upregulates VEGFA expression and promotes angiogenesis by activating the NF-κB pathway	(39)
	Colorectal Cancer	Promotes tumor angiogenesis and metastasis through activation of the AKT1/mTOR/VEGFA signaling pathway	(40)
	Breast Cancer	Inhibits tumor growth by suppressing VEGF expression	(11)
	Clear Cell Renal Cell Carcinoma	Promotes angiogenesis through the Tie-2 pathway	(41)
Angiogenic Mimicry	NSCLC	Promotes VM formation in NSCLC cells through the PI3K/AKT signaling pathway	(42)
	Hepatocellular Carcinoma	Promotes VM formation by activating the PI3K/AKT/MMPs pathway and upregulating the expression of MMP2, MMP14, VE-cadherin, and vimentin during EMT process	(43)

B7-H3, B7 homolog 3; Jak2, Janus kinase 2; TM4SF1, transmembrane−4 L6 family member 1; SIRT1, sirtuin 1;Nrf2 and its target genes, increasing protein levels of HIF1α, hypoxia−inducible factor−1α; LDHA, lactate dehydrogenase A; PDK1, phosphoinositide-dependent protein kinase-1; PFKFB3, 6-Phosphofructo 2-kinase/fructose 2, 6-bisphosphatase 3;VM, vasculogenic mimicry.

### 2.2 Proliferation, invasion, migration

Recent studies have revealed the significant role of B7-H3 in promoting cancer cell proliferation, migration, and invasion (21, 35, 44, 45). In various cancers, including oral cancer, hepatocellular carcinoma, colorectal cancer, lung adenocarcinoma, hematologic malignancies, and gynecologic tumors, B7-H3 has been found to promote cancer cell proliferation by regulating different signaling pathways (46). B7-H3 can also enhance cancer cell migration by modulating the expression of E-cadherin, increasing their migratory capacity (20). Additionally, B7-H3 can augment cancer cell invasion into surrounding tissues by regulating the activity of matrix metalloproteinases (MMPs) (22). These findings provide us with new insights and suggest that targeting B7-H3 and its associated signaling pathways could be an effective strategy for the treatment of these cancers. The summary of signaling pathways mediating the proliferation, invasion, and migration effects of B7-H3 is presented in **Table 1**.

### 2.3 Drug resistance

Drug resistance is a reflection of tumor evolution and is a major cause of cancer recurrence and patient mortality (47). Studies have shown that overexpression of B7-H3 often leads to drug resistance in cancer cells. Different mechanisms of resistance exist in different tumor cells. For example, the high expression of the B7-H3 immune checkpoint protein in neuroblastoma is involved in carcinogenic signaling, tumor cell plasticity, and the development of drug resistance (48). However, inhibiting the expression of B7-H3 or blocking its associated signaling pathways can reverse this resistance. One approach is to use a combination of paclitaxel and MEK inhibitors to block the B7-H3 signal, which has been demonstrated to effectively reverse chemotherapy resistance (16). Another approach is to use the specific inhibitor LY294002 to block the PI3K signaling pathway, which can also effectively inhibit this resistance (49). Another study found that bromodomain and extra-terminal domain inhibitors could be used to reduce the levels of B7-H3 protein and mRNA in pancreatic cancer cells, providing a new therapeutic strategy to overcome immune and chemotherapy resistance in pancreatic cancer (21). In related studies on traditional Chinese medicine, researchers found that artemisinin-mediated inhibition of B7-H3 may help enhance neuroblastoma cell sensitivity to doxorubicin (50). Additionally, Astragaloside IV enhanced the chemosensitivity to cisplatin by inhibiting B7-H3, suggesting that a combination therapy using Astragaloside IV and a B7-H3 inhibitor may be a potential treatment method for lung cancer patients (51). The summary of signaling pathways mediating the drug resistance effects of B7-H3 is presented in **Table 1**.

### 2.4 Radiation resistance

Radiation therapy is a common treatment modality for solid tumors; however, radiation resistance is a major factor contributing to treatment failure in cancer (52). A study by Ma et al. reported an interesting phenomenon: after X-ray irradiation, the expression of B7-H3 was upregulated in colorectal cancer cells, and this upregulation depended on the upregulation of KIF15 via the NF-κB signaling pathway. Further research revealed that B7-H3/KIF15, through activation of the ERK1/2 signaling pathway, promoted radiation resistance in colorectal cancer (31). Additionally, Zhou et al. found that B7-H3 increased the radiation resistance of gastric cancer cells by regulating baseline levels of cellular autophagy. In cells with high B7-H3 expression, increasing the baseline level of cellular autophagy using rapamycin enhanced their sensitivity to radiation (32). These studies all point to the important role of B7-H3 in regulating tumor cell resistance to radiation therapy. Therefore, finding effective ways to inhibit or modulate the expression and function of B7-H3 may provide new strategies to improve the effectiveness of radiation therapy.

### 2.5 Metabolism

The Warburg effect is one of the characteristic features of tumor cell metabolism, where cancer cells tend to produce energy through glycolysis even in the presence of abundant oxygen, which is significantly different from the metabolic mechanism of normal cells (53). Research by Lim et al. found that B7-H3 plays an important role in the metabolic reprogramming of cancer cells. In cells expressing B7-H3, glucose uptake and lactate production are increased (34). On the other hand, there is an interaction between the glycolytic enzyme ENO1 and B7-H3. Downregulation of B7-H3 in cervical cancer HeLa cells leads to decreased levels of ATP, lactate, c-Myc, and lactate dehydrogenase A (54). Furthermore, overexpression of B7-H3 effectively increases glucose consumption and lactate production rate, while knockout of B7-H3 has the opposite effect. Liu et al. demonstrated that using the anti-B7-H3 antibody 8H9 can shift cellular metabolism from glycolysis to oxidative phosphorylation, and they suggested that anti-B7-H3 blockade may alter tumor glucose metabolism through reactive oxygen species-mediated pathways by measuring cellular ROS levels in A549 cells treated with the anti-B7-H3 antibody using fluorescence probes (55). In addition to changes in glucose metabolism, cancer cells often possess altered fatty acid metabolism properties. In glioma cells, genetic risk features associated with fatty acid catabolism are significantly correlated with the immune checkpoint molecule B7-H3 (56). These research findings further emphasize the important role of B7-H3 in the metabolic regulation of cancer cells and may provide new therapeutic strategies for targeting this process. The summary of signaling pathways mediating the metabolism effects of B7-H3 is presented in **Table 1**.

## 3 The role of B7-H3 in tumor vasculature and clinical treatment

### 3.1 The role of B7-H3 in tumor vasculature

In cancer development, angiogenesis plays a crucial role by providing oxygen and nutrients to tumors while allowing their spread to other parts of the body. Thus, inhibiting or reducing the blood supply in the tumor microenvironment to restrict tumor angiogenesis has become a key strategy against various solid tumors (57). B7-H3 is a highly expressed protein in many types of cancer, including lung, breast, colorectal, endometrial, renal, ovarian, hepatocellular carcinoma, and melanoma. However, this protein is not expressed in normal vascular tissues (4, 5). Furthermore, in hepatocellular carcinoma, colorectal cancer, renal cell carcinoma, and melanoma, the expression rate of B7-H3 in the tumor vasculature reaches 86% to 98% (58). In ovarian cancer, although the expression rate of B7-H3 is only 44%, it is undetectable in normal ovarian vascular tissues (5).

Under normal physiological conditions, the process of angiogenesis is strictly regulated by factors that promote and inhibit angiogenesis to maintain a stable internal environment and prevent uncontrolled blood vessel growth. However, B7-H3 protein can disrupt this balance by enhancing the activity of several pro-angiogenic factors, favoring tumor development (9). It mainly activates multiple signaling pathways, such as MMP-2, MMP-9, NF-κB, AKT1/mTOR/VEGFA, and Tie-2, thus promoting tumor angiogenesis and further facilitating tumor growth and spread (38–41). By inhibiting key molecules in these signaling pathways, we can effectively inhibit angiogenesis. For example, in neuroblastoma, overexpression of miR-29 can activate the JAK/STAT1 signaling pathway, weakening the regulatory effect of MYC-B7-H3 and inhibiting tumor angiogenesis (38). Additionally, Wang et al. found that recombinant VEGFA can eliminate the inhibitory effect of shB7-H3 on human umbilical vein endothelial cell angiogenesis in conditioned medium from colorectal cancer cells. Furthermore, siRNA against VEGFA or neutralizing antibodies against VEGFA can reverse the effects of B7-H3 in overexpressed colorectal cancer cell-conditioned medium on human umbilical vein endothelial cell angiogenesis (39). These studies further elucidate the potential role of B7-H3 in tumor angiogenesis and may serve as a crucial target for future anti-angiogenic therapies.

Moreover, B7-H3 also plays an important role in vasculogenic mimicry (VM). VM is an alternative microcirculation pattern independent of angiogenesis, where cancer cells form microvascular-like channels to provide nutrients and oxygen to tumors (59). Recent studies have found that B7-H3 expressed in tumors promotes VM formation in non-small cell lung cancer cells through the PI3K/AKT signaling pathway without affecting normal angiogenesis, providing a new strategy for future anticancer therapies (42). The summary of signaling pathways mediating B7-H3-induced tumor angiogenesis is presented in **Table 1**.

### 3.2 Clinical treatment

In numerous types of tumors, the widespread overexpression of B7-H3 on cancer cells and tumor-associated blood vessels makes it a potential ideal dual-target for therapy. CD276-ADC, utilizing pyrrolobenzodiazepine as a linker, demonstrates significant cytotoxicity against cancer cells while also exhibiting remarkable destructive effects on the tumor vascular system. This therapy shows great potential in eradicating large existing tumors and metastatic lesions, with the possibility of significantly improving long-term overall survival rates for patients (5). To target the overexpression of B7-H3 in tumor cells and tumor vasculature, along with its limited expression in normal tissue vasculature, researchers have employed an innovative strategy by conjugating the Fab fragment of anti-CD276 antibody with the photosensitizer IRDye700 to form a novel CD276-targeted agent. The findings show that combining photodynamic therapy using this agent with inhibition strategies targeting the PD-L1/PD-1 axis can induce strong local and systemic anti-tumor responses, effectively eliminating primary and metastatic tumors (60). Chen et al. reported the development of pH-responsive drug release using B7H3-targeted doxorubicin-conjugated gold nano-cages (B7H3/Dox@GNCs), which represents a selective, precise, and synergistic chemotherapy-photothermal therapy for NSCLC, capable of simultaneously destroying B7H3-positive tumor cells, tumor-associated vasculature, and cancer-associated fibroblasts (61). The expression of B7-H3 in the tumor vasculature can also be utilized to significantly improve the diagnostic accuracy of breast cancer, which is undoubtedly an important discovery (62).

Furthermore, as a promising immunotherapy target, B7-H3 has been applied in combination treatment strategies. When used as a key element in T cell co-stimulation and anti-angiogenesis therapy for hepatocellular carcinoma, the effect of B7-H3 is significantly superior to using these treatments alone. Injecting plasmids expressing B7-H3 into subcutaneous tumors in mice resulted in complete eradication of these tumors within 24 hours, which was not achieved when using angiostatin or B7-H3 treatment alone (63). This process revealed the ability of angiostatin gene transfer to inhibit tumor angiogenesis and enhance NK cell infiltration, as well as the critical role of B7-H3 in activating CD8^+^ tumor infiltration, increasing circulating IFN-c levels, and even completely regressing distant tumor nodules (63). However, although B7-H3 plays an important role in the aforementioned combination treatment strategies, its primary role in the broader field of immunotherapy is still as an immune checkpoint inhibitor. For example, in the treatment of triple-negative breast cancer, Cheng et al. found that blocking B7-H3 with anti-B7-H3 antibodies improved the abnormal state of the tumor vascular system, thereby enhancing the effects of chemotherapy and PD-1 treatment (64). These research findings further emphasize that B7-H3 can serve as an important therapeutic target in anticancer treatment strategies, whether used alone or in combination with other treatment modalities. More clinical trials targeting B7-H3 for cancer treatment are listed in **Table 2**.

**Table 2:** Clinical Trials Targeting B7-H3.

Intervention type	Intervention	Trial number	Phase	Cancer type
ADCC	MGA271	NCT01918930	I	Melanoma
		NCT02982941	I	Neuroblastoma Rhabdomyosarcoma Osteosarcoma
		NCT01391143	I	Prostate Cancer, Melanoma, Renal Cell Carcinoma, Triple-negative Breast Cancer, Head and Neck Cancer, Bladder Cancer, Non-small Cell Lung Cancer
		NCT0298294	I	Neuroblastoma, Rhabdomyosarcoma, Osteosarcoma, Ewing Sarcoma, Wilms Tumor, Desmoplastic Small Round Cell Tumor
		NCT02923180	II	Prostate Cancer
	DS-5573a	NCT02192567	I	Advanced Solid Malignant Tumors
ADC	MGC018	NCT03729596	I/II	Advanced Solid Malignant Tumors
	I-DXd	NCT05280470	II	Extensive-stage Small-cell Lung Cancer
		NCT06330064	II	Recurrent or Metastatic Solid Tumors
		NCT06203210	III	Small Cell Lung Cancer
		NCT06362252	Ib/II	Extensive Stage-small Cell Lung Cancer
	HS-20093	NCT06112704	II	Advanced Solid Tumors
		NCT06332170	I	Advanced Solid Tumors
		NCT05830123	II	Osteosarcoma Sarcoma
		NCT05276609	I	Advanced Solid Tumors
		NCT06001255	II	Metastasis Castration Resistant Prostate Cancer(mCRPC)
		NCT06007729	II	Head and Neck Squamous Cell Carcinoma
	BAT8009	NCT05405621	I	Locally Advanced/Metastatic Solid Tumours
	DS-7300a	NCT04145622	I/II	Advanced Solid Tumor Malignant Solid Tumor
B7-H3 X CD3 BiAb	MGD009	NCT02628535	I	13 Types of Tumors
CAR-T	B7-H3-CAR T cells	NCT04897321	I	15 Types of Tumors
		NCT05835687	I	7 Types of Central Nervous System Tumors
		NCT04185038	I	10 Types of Central Nervous System Tumors
		NCT04670068	I	Epithelial Ovarian Cancer
		NCT05474378	I	Brain and Nervous System
		NCT05366179	I	Glioblastoma Multiforme
		NCT05190185	I	Malignant Melanoma, Lung Cancer, or Colorectal Cancer
		NCT04385173	I	Recurrent Glioblastoma Refractory Glioblastoma
		NCT04077866	I/II	Recurrent Glioblastoma Refractory Glioblastoma
		NCT05241392	I	Glioblastoma
		NCT04864821	Early Phase 1	Osteosarcoma, Neuroblastoma, Gastric Cancer, Lung Cancer
		NCT05143151	I/II	Advanced Pancreatic Carcinoma
		NCT05562024	I	B7-H3-positive Relapsed/Refractory Neuroblastoma
		NCT06221553	Early Phase 1	DIPG Brain Tumor Diffuse Intrinsic Pontine Glioma
	second generation 4–1BBζ B7H3-EGFRt-DHFR	NCT04483778	I	16 Types of Pediatric Solid Tumors
	SC- CAR 4BRAIN	NCT05768880	I	Malignant Central Nervous System Neoplasm
	B7-H3 UCAR-T cell	NCT05752877		Advanced Glioma Complication of Chimeric Antigen Receptor (CAR-T) Cell Therap
	KT095 CAR-T cell	NCT05515185	Early Phase 1	Advanced Solid Tumor
	B7-H3 and 11 other engineered CAR-T	NCT04842812	I	Liver CancerLung CancerBreast CancerColo-rectal CancerBrain TumorSolid Tumor, AdultPD1CTLA4
	4SCAR-276	NCT04432649	I/II	Solid Tumor
	fhB7H3.CAR-Ts	NCT05211557	I/II	Ovarian Cancer
	EGFR/B7H3 CAR-T	NCT05341492	Early Phase 1	EGFR/B7H3-positive Advanced Lung/Breast Cancer
	B7-H3 and 10 other CAR-T	NCT03198052	I	Lung Cancer
	iC9-CAR.B7-H3 T cells	NCT06305299	I	Ovary Neoplasm Ovarian Cancer Epithelial Ovarian Cancer
		NCT06347068	I	Breast Cancer Relapse Resistant Cancer
	4SCAR-T cells	NCT04637503	I/II	Neuroblastoma
CAR-γδ T	B7-H3-CAR -γδ T cells	NCT06018363		Brain Gliomas
CAR-gd T	B7-H3-CAR -gd T cells	NCT05731219		Relapsed/Refractory Acute Myeloid Leukemia
		NCT05722171		Relapsed/Refractory Acute Myeloid Leukemia
Radioimmunotherapy	124I-omburtamab	NCT01502917	I	Brain Cancer Brain Stem Glioma
	177Lu-DTPA-omburtamab	NCT04167618	I/II	Medulloblastoma, Childhood
		NCT04315246	I/II	Leptomeningeal Metastasis Solid Tumor, Adult
	131I-omburtamab	NCT00089245	I	Brain and Central Nervous System Tumors, Neuroblastoma, Sarcoma
		NCT00582608	Not Applicable	CNS Cancer, Neuroblastoma, Sarcoma
		NCT01099644	I	Peritoneal Cancer
		NCT03275402	II/III	Neuroblastoma, CNS Metastases, Leptomeningeal Metastases
		NCT04022213	II	Desmoplastic Small Round Cell Tumor, Peritoneal Cancer, Peritoneal Carcinoma
		NCT05064306		Central Nervous, System/Leptomeningeal, Neoplasms
		NCT04743661	II	Recurrent Medulloblastoma Recurrent Ependymoma
		NCT05063357	I	DIPG
Anti-B7-H3 ADCC with Anti-PD-1 Ab	MGA271/pembrolizumab/MGA 012	NCT02475213	I	Melanoma, Head and Neck Cancer, Non Small Cell Lung Cancer, Urethelial Carcinoma
	Enoblituzumab MGA012 MGD013	NCT04129320	II/III	Head and Neck Cancer, Squamous Cell Carcinoma of Head and Neck
Anti-B7-H3 ADCC with Anti-PD-1 Ab or PD-1 X LAG-3 BiAb	MGA271/MGA012/MGD013	NCT04634825	II	Head and neck cancer
Anti-B7-H3 ADC with Anti-PD-1 Ab	MGC018/MGA012	NCT03729596	I/II	6 advanced solid tumors
B7-H3 CD3 BiAb with Anti-PD-1 Ab	MGD009/MGA012	NCT03406949	I	Advanced Solid Tumors
Anti-B7-H3 ADC with CD19 X CD3 BiAb	MGC018/MGD019	NCT05293496	I	7 advanced solid tumors
Anti-B7-H3 ADCC with NK cell enhancing	MGA271/FT516 and IL2	NCT04630769	I	Ovarian Cancer, Fallopian Tube Adenocarcinoma, Primary Peritoneal Cavity Cancer
Anti-B7-H3 ADCC with Anti-CTLA-4 Ab	enoblituzumab plus ipilimumab	NCT02381314	I	Melanoma, Non Small Cell Lung Cancer

## 4 Conclusion

Compared to other immune checkpoints, the uniqueness of B7-H3 lies in its ability to regulate both innate and adaptive immune responses. Furthermore, it promotes tumor development by stimulating various non-immune-related functions, making it an important prognostic marker for adverse outcomes. B7-H3 influences the life processes of tumor cells, including apoptosis, proliferation, invasion, migration, drug resistance, resistance to radiotherapy, metabolic processes, and abnormal angiogenesis, through the regulation of various signaling pathways. Therefore, inhibiting B7-H3 expression or blocking related signaling pathways may be an effective approach to prevent tumor progression.

Considering the high expression of B7-H3 in tumor cells and tumor vasculature, along with its low expression in normal vasculature, it becomes an ideal dual-target in cancer therapy. By considering B7-H3 as a bridge between tumor cells and the tumor vascular system, we can achieve dual therapeutic effects by targeting B7-H3. However, since the role and signaling pathways of B7-H3 may vary among different tumors and even within the vascular systems of different tumors, gaining a deeper understanding of the specific functions of B7-H3 in particular tumors will help us design more targeted treatment plans for patients, thus effectively controlling tumor development. This could be an important direction for future research in cancer therapy.

## Author contributions

SW: Writing – original draft, Writing – review & editing. CH: Visualization, Writing – review & editing. KH: Visualization, Writing – review & editing. XJ: Conceptualization, Writing – review & editing.
